# Phase 1b/2a study of trastuzumab emtansine (T-DM1), paclitaxel, and pertuzumab in HER2-positive metastatic breast cancer

**DOI:** 10.1186/s13058-016-0691-7

**Published:** 2016-03-15

**Authors:** Ian E. Krop, Shanu Modi, Patricia M. LoRusso, Mark Pegram, Ellie Guardino, Betsy Althaus, Dan Lu, Alexander Strasak, Anthony Elias

**Affiliations:** Breast Oncology, Dana-Farber Cancer Institute, 450 Brookline Avenue, Boston, MA 02215 USA; Memorial Sloan Kettering Cancer Center, 1275 York Avenue, New York, NY 10065 USA; Weill Cornell Medical College, 445 E 69th St, New York, NY 10021 USA; Yale University, 800 Howard Ave, New Haven, CT 06519 USA; Stanford Cancer Institute, Stanford University School of Medicine, 900 Blake Wilbur, Stanford, CA 94305 USA; Genentech, Inc., 1 DNA Way, South San Francisco, CA 94080 USA; F. Hoffmann-La Roche Ltd., Grenzacherstrasse 124, Basel, ch-4070 Switzerland; University of Colorado Cancer Center, 1665 Aurora Ct, Aurora, CO 80045 USA

## Abstract

**Background:**

In pre-clinical studies, the anti-tumor activity of T-DM1 was enhanced when combined with taxanes or pertuzumab. This phase 1b/2a study evaluated the safety/tolerability of T-DM1 + paclitaxel ± pertuzumab in HER2-positive advanced breast cancer.

**Methods:**

In phase 1b (n = 60), a 3 + 3 dose-escalation approach was used to determine the maximum tolerated dose (MTD) of T-DM1 + paclitaxel ± pertuzumab. The primary objective of phase 2a was feasibility, with 44 patients randomized to T-DM1 + paclitaxel ± pertuzumab at the MTD identified in phase 1b.

**Results:**

The MTD was T-DM1 3.6 mg/kg every three weeks (q3w) or 2.4 mg/kg weekly + paclitaxel 80 mg/m^2^ weekly ± pertuzumab 840 mg loading dose followed by 420 mg q3w. Phase 2a patients had received a median of 5.0 (range: 0–10) prior therapies for advanced cancer. In phase 2a, 51.2 % received ≥12 paclitaxel doses within 15 weeks, and 14.0 % received 12 paclitaxel doses by week 12. Common all-grade adverse events (AEs) were peripheral neuropathy (90.9 %) and fatigue (79.5 %). A total of 77.3 % experienced grade ≥3 AEs, most commonly neutropenia (25.0 %) and peripheral neuropathy (18.2 %). Among the 42 phase 2a patients with measurable disease, the objective response rate (ORR) was 50.0 % (95 % confidence interval (CI) 34.6–65.4); the clinical benefit rate (CBR) was 56.8 % (95 % CI 41.6–71.0). No pharmacokinetic interactions were observed between T-DM1 and paclitaxel.

**Conclusions:**

This regimen showed clinical activity. Although there is potential for paclitaxel to be added to T-DM1 ± pertuzumab, peripheral neuropathy was common in this heavily pretreated population.

**Trial registration:**

ClinicalTrials.gov NCT00951665. Registered August 3, 2009.

**Electronic supplementary material:**

The online version of this article (doi:10.1186/s13058-016-0691-7) contains supplementary material, which is available to authorized users.

## Background

Trastuzumab emtansine (T-DM1) is a human epidermal growth factor receptor 2 (HER2)–targeted antibody–drug conjugate composed of the humanized monoclonal antibody trastuzumab conjugated via a stable linker to the cytotoxic microtubule polymerization inhibitor DM1 [1]. In the phase 3 EMILIA trial of patients with metastatic breast cancer (MBC) previously treated with trastuzumab and a taxane, single-agent T-DM1 was associated with statistically significantly improved progression-free survival (PFS; 9.6 vs. 6.4 months; hazard ratio [HR], 0.65; *P* < 0.001) and overall survival (OS; 30.9 vs. 25.1 months; HR, 0.68; *P* < 0.001) relative to lapatinib plus capecitabine [2]. In the phase 3 TH3RESA study of patients with MBC previously administered ≥ 2 HER2-targeted therapies in the advanced disease setting and a taxane in any setting, single-agent T-DM1 led to a statistically significant improvement in PFS vs. treatment of physician’s choice (6.2 vs. 3.3 months; HR, 0.53; *P* < 0.0001) [3]. In a phase 2 trial of first-line MBC, T-DM1 was associated with significantly longer PFS than trastuzumab + docetaxel (14.2 vs. 9.2 months; HR, 0.59; *P* = 0.035) [4].

Taxanes are mainstay chemotherapy for the treatment of breast cancer [5], and paclitaxel is used in combination with trastuzumab for HER2-positive MBC. Pertuzumab is a humanized monoclonal antibody that recognizes a binding domain on HER2 distinct from that of trastuzumab [6–10]. In the phase 3 CLEOPATRA trial of HER2-positive MBC, the combination of pertuzumab, trastuzumab, and docetaxel significantly prolonged PFS (median 18.5 vs. 12.4 months; HR, 0.62; *P* < 0.001) [11] and OS (median 56.5 months vs. 40.8 months: HR, 0.68, *P* < 0.001) [12] vs. trastuzumab + docetaxel.

In pre-clinical experiments, the antitumor activity of T-DM1 was enhanced when combined with paclitaxel [13] or pertuzumab [14]. Based on these promising pre-clinical data, this open-label, multicenter, phase 1b/2a study (TDM4652g/NCT00951665) was designed to investigate the feasibility of combination treatment with T-DM1 + paclitaxel ± pertuzumab in HER2-positive locally advanced breast cancer (LABC) or MBC.

Here we show that T-DM1 + paclitaxel ± pertuzumab shows marked clinical activity in patients with previously treated HER2-positive LABC or MBC, although peripheral neuropathy was a common adverse event (AE).

## Methods

### Study design

**Fig. 1:**
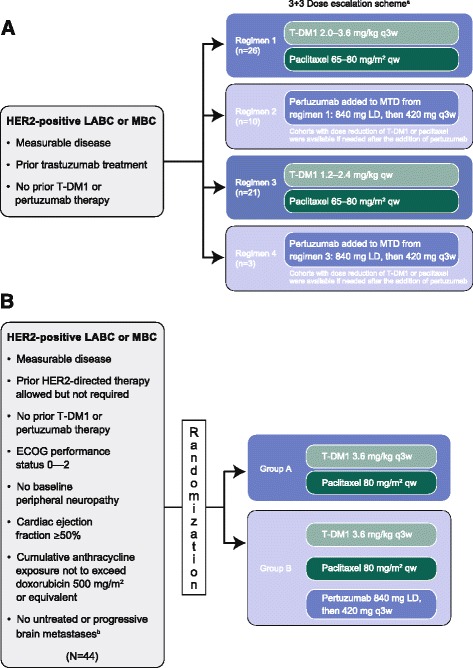
Design of (**a**) phase 1b and (**b**) phase 2a. ^a^MTD is the highest dose at which 0/3 patients or 1/6 patients experienced a dose-limiting toxicity. ^b^Brain metastases that have required any type of therapy to control symptoms in the 60 days prior to first study treatment. *ECOG* Eastern Cooperative Oncology Group, *HER2* human epidermal growth factor receptor 2, *LABC* locally advance breast cancer, *LD* loading dose, *MBC* metastatic breast cancer, *MTD* maximum tolerated dose, *qw* weekly, *q3w* every three weeks, *T-DM1* trastuzumab emtansine

The phase 1b dose-finding portion of this phase 1b/2a study evaluated the efficacy, safety, tolerability, and pharmacokinetics of T-DM1 + paclitaxel ± pertuzumab (Fig. 1a). A traditional 3 + 3 dose-escalation approach was used to explore four regimens. Under regimen 1, intravenous T-DM1 was administered every three weeks (q3w) at 2.0, 2.4, 3.0, or 3.6 mg/kg, and intravenous paclitaxel was administered weekly at 65 mg/m^2^ or 80 mg/m^2^. The maximum tolerated dose (MTD) of this combination was utilized in regimen 2, with the addition of intravenous pertuzumab (loading dose of 840 mg on day 1 of cycle 1 followed by 420 mg q3w in subsequent cycles). Under regimen 3, weekly T-DM1 1.2, 1.6, 2.0, or 2.4 mg/kg was administered in combination with weekly paclitaxel 65 mg/m^2^ or 80 mg/m^2^. The MTD of this combination was used in regimen 4, with the addition of pertuzumab (dose as above). Patients were followed for a minimum of 22–23 days before additional patients were enrolled to the next dose cohort. Patients were assessed for dose-limiting toxicities (DLTs) during cycle 1 (Additional file 1: Table S1); the minimum DLT observation period was 23 days for regimens 1 and 3, and 22 days for regimens 2 and 4. Based on DLTs observed on-study, DLT criteria were revised to establish a more clinically relevant MTD (Additional file 1: Table S1). Using the MTDs identified in phase 1b, phase 2a patients (Fig. 1b) were randomized (1:1) via an interactive voice response system to T-DM1 3.6 mg/kg q3w + weekly paclitaxel 80 mg/m^2^ (Group A) or to T-DM1 3.6 mg/kg q3w + weekly paclitaxel 80 mg/m^2^ + pertuzumab (dose described) (Group B).

In both study phases, treatment continued until disease progression or unacceptable toxicity. In phase 2a, paclitaxel could be discontinued after 12 doses at the investigator’s discretion for reasons other than disease progression or unacceptable toxicity. Patients who discontinued paclitaxel could continue T-DM1 ± pertuzumab in the absence of disease progression. Dose delays of up to 21 days were allowed for T-DM1, paclitaxel, and pertuzumab.

Patients who developed isolated brain metastases could receive central nervous system radiotherapy and resume study treatment if systemic disease was controlled and ≤1 treatment cycle was missed. An Eastern Cooperative Oncology Group (ECOG) performance status of 0–2 was required to continue therapy. Patients with ongoing clinical benefit, acceptable toxicity, and adequate cardiac function could receive treatment for up to one year. After one year of treatment, patients without disease progression had the option to enroll in an ongoing extension study (TDM4529g/NCT00781612). To allow completion of the present study, phase 2a patients could enroll in TDM4529g 16 weeks after the last patient enrolled in TDM4652g.

This study was reviewed and approved by the relevant institutional review board/ethics committee at each study site (Dana-Farber Cancer Institute Institutional Review Board, Colorado Multiple Institutional Review Board, Western Institutional Review Board Panel 7, Memorial Sloan Kettering Cancer Center Institutional Review Board, Wayne State University Institutional Review Board, or Stanford University Research Compliance Office), and was conducted in accordance with the Declaration of Helsinki, Good Clinical Practice guidelines, and applicable laws. All patients provided written informed consent.

### Patients

Eligible patients were aged ≥18 years with an ECOG performance status of 0–2 and measurable or evaluable HER2-positive (immunohistochemistry 3+, fluorescence in situ hybridization–positive, or chromogenic in situ hybridization–positive by local assessment) unresectable LABC or MBC. Phase 1b patients had received prior treatment with trastuzumab in any line, but this was not a requirement for phase 2a. A cardiac ejection fraction ≥50 % by echocardiogram or multigated acquisition (MUGA) scan, adequate hematologic and end-organ function, and life expectancy ≥90 days as assessed by the investigator were also required. Exclusion criteria are described in Additional file 2.

### Study objectives

The primary objectives of phase 1b were to determine MTD, identify DLTs, and characterize the safety, tolerability, and pharmacokinetics of T-DM1 (q3w and weekly) + paclitaxel ± pertuzumab. The primary objectives of phase 2a were to further characterize the safety and feasibility of T-DM1 + paclitaxel ± pertuzumab and assess the proportion of patients able to receive 12 doses of T-DM1 + paclitaxel ± pertuzumab. Secondary objectives of both study phases included overall response rate (ORR), clinical benefit rate (CBR), PFS, and duration of response. Biomarkers were an exploratory endpoint.

### Assessments

Pharmacokinetic and biomarker analyses were performed, as described in the supplemental materials (Additional file 2). Patients were monitored for AEs and serious AEs throughout the study and for 30 days after last treatment dose. Investigators were instructed to follow unresolved AEs and serious AEs until event resolution/stabilization, patient was lost to follow-up, or study treatment/participation was determined to not be the cause of the event. Echocardiogram or MUGA scans were performed at screening and the end of cycle 2. Scans were repeated every three cycles thereafter throughout phase 1b and every four cycles thereafter throughout phase 2a. Patients who discontinued T-DM1 because of AEs were followed for 30 days for safety and every six weeks for disease progression until the initiation of another anticancer therapy or study withdrawal. AEs were graded according to National Cancer Institute Common Terminology Criteria for AEs v.3.

The feasibility of T-DM1 + paclitaxel ± pertuzumab was assessed by the percentage of patients who completed 12 consecutive weeks of paclitaxel and the percentage who completed ≥12 paclitaxel doses within the first 15 weeks of the study. Patients who discontinued paclitaxel due to disease progression before completing 12 doses were not evaluable for feasibility. The percentage of patients who completed ≥8 paclitaxel doses within the first 12 weeks of the study was analyzed post hoc.

Radiologic tumor assessments, graded according to modified Response Evaluation Criteria in Solid Tumors (RECIST) v1.0 [15], were performed at screening, the end of cycle 2, and every two cycles thereafter throughout phase 1b. For phase 2a, radiologic tumor assessments were performed at baseline and every four cycles (12 weeks) thereafter. Investigator-assessed ORR was based on modified RECIST v1.0; responses were confirmed. CBR was defined as the percentage of patients with investigator-assessed complete response, partial response, or stable disease of ≥6 months duration.

### Statistical analyses

All statistical analyses are descriptive (SAS® version 9.2). Patients who received ≥1 dose of study treatment were included in the safety and efficacy analyses. Patients who discontinued treatment due to disease progression before completing 12 doses within 15 weeks were not included in the feasibility assessment. Kaplan–Meier methodology was used to estimate median PFS; corresponding 95 % confidence intervals were determined via the Clopper-Pearson method. The cut-off for these analyses was 15 August 2013, and does not include data from the extension study.

## Results

### Patients

Sixty patients were enrolled to phase 1b (regimen 1, n = 26; regimen 2, n = 10; regimen 3, n = 21; regimen 4, n = 3) (Fig. 1a), and 44 were randomized to phase 2a (Group A, n = 22; Group B, n = 22). The demographic and baseline characteristics of the phase 1b and 2a populations are shown in Table 1; some imbalances were seen in the cohort of patients randomized to phase 2a. Compared with Group B, fewer patients in Group A were hormone receptor–positive (45.5 % vs. 59.1 %), anthracycline-exposed (59.1 % vs. 77.3 %), and white (72.7 % vs. 90.9 %), while more had been previously administered taxanes (90.9 % vs. 72.7 %).

**Table 1:** Demographic and baseline characteristics

Characteristic	Phase 1b	Phase 2a		
	All patients (N = 60)	Group A (n = 22)	Group B (n = 22)	All patients (N = 44)
Median age, years (range)	53.5 (23–77)	50.0 (35–81)	54.0 (43–72)	52.5 (35–81)
Gender, n (%)				
Female	58 (97)	21 (95.5)	22 (100)	43 (97.7)
Male	2 (3)	1 (4.5)	0	1 (2.3)
Race, n (%)				
White	55 (92)	16 (72.7)	20 (90.9)	36 (81.8)
Asian	3 (5)	2 (9.1)	0	2 (4.5)
Black	1 (2)	3 (13.6)	1 (4.5)	4 (9.1)
Not available	1 (2)	1 (4.5)	1 (4.5)	2 (4.5)
ECOG performance status, n (%)				
0	28 (47)	12 (54.5)	14 (63.6)	26 (59.1)
1	29 (48)	8 (36.4)	7 (31.8)	15 (34.1)
2	3 (5)	2 (9.1)	1 (4.5)	3 (6.8)
Hormone status, n (%)				
ER+ and/or PR+	43 (61)	10 (45.5)	13 (59.1)	23 (52.3)
ER- and PR–	16 (27)	12 (54.5)^a^	9 (40.9)	21 (47.7)
Unknown	1 (2)	0	0	0
Prior radiotherapy, n (%)	47 (78)	17 (77.3)	17 (77.3)	34 (77.3)
Prior surgery, n (%)	60 (100)	22 (100)	22 (100)	44 (100)
Prior systemic therapies, n (%)	60 (100)	22 (100)	22 (100)	44 (100)
Trastuzumab	60 (100)	22 (100)	21 (95.5)	43 (97.7)
Chemotherapy	60 (100)	22 (100)	22 (100)	44 (100)
Anthracycline	47 (78)	13 (59.1)	17 (77.3)	30 (68.2)
Taxane	54 (90)	20 (90.9)	16 (72.7)	36 (81.8)
Lapatinib	48 (80)	15 (68.2)	14 (63.6)	29 (65.9)
Hormonal	39 (65)	12 (54.5)^a^	13 (59.1)	25 (56.8)^a^
Experimental		7 (31.8)	5 (22.7)	12 (27.3)
Other biologic		3 (13.6)	1 (4.5)	4 (9.1)
Number of prior systemic agents, median (range)^b^	8.5 (2–21)	7.5 (2–12)	6.5 (3–14)	7.0 (2–14)
Number of prior systemic agents in the metastatic setting, median (range)^b^	6.5 (0–18)	6.0 (1–9)	5.0 (0–10)	5.0 (0–10)^c^
Median time since metastatic diagnosis, months (range)	37.9 (4–111)	46.9 (11–199)	48.1 (1–170)	46.9 (1–199)

*ECOG* Eastern Cooperative Oncology Group, *ER* estrogen receptor, *PR* progesterone receptor

^a^Two patients with hormone receptor-negative disease received prior hormonal therapy

^b^Does not include hormonal therapy

^c^One patient did not receive any prior systemic agents in the metastatic setting

Forty-one (68.3 %) phase 1b patients withdrew from the study due to disease progression (n = 29), AEs (n = 4), death (n = 3), physician decision (n = 4), and patient decision (n = 1). Nineteen (43.2 %) phase 2a patients discontinued because of disease progression (n = 11), progressive disease (PD)–related death (n = 2), AEs (n = 2), patient decision (n = 2), physician decision (n = 1), and loss to follow up (n = 1).

### Maximum tolerated dose

Thirty of the 60 (50.0 %) phase 1b patients were assessed using the original DLT criteria. The following DLTs were observed: neutropenia (n = 3), increased alanine aminotransferase (n = 2), increased aspartate aminotransferase (n = 1), thrombocytopenia (n = 1), and dehydration (n = 1). Following revision of the DLT criteria (Additional file 1: Table S1), no additional DLTs were reported. The MTDs were identified as T-DM1 3.6 mg/kg q3w or weekly 2.4 mg/kg + weekly paclitaxel 80 mg/m^2^ and pertuzumab 840 mg loading dose followed by 420 mg q3w. The addition of pertuzumab did not alter the MTDs for T-DM1 + paclitaxel.

### Safety

Across both study phases, the most common all-grade AEs were peripheral neuropathy (phase 1b, 90.0 %; phase 2a, 90.9 %) and fatigue (phase 1b, 85.0 %; phase 2a, 79.5 %) (Table 2, Additional file 3: Table S2). In phase 1b, 80.0 % (48/60) experienced ≥1 grade ≥3 AE, most commonly peripheral neuropathy (23.3 %), neutropenia (20.0 %), and fatigue (18.3 %) (Additional file 3: Table S2). Of the 44 phase 2a patients, 77.3 % experienced ≥1 grade ≥3 AE, most commonly neutropenia (25.0 %), peripheral neuropathy (18.2 %), and thrombocytopenia (15.9 %) (Table 2). Within the phase 2a cohort the incidence of common all-grade AEs was generally similar without (Group A) or with pertuzumab (Group B); however, rates of all-grade dry eye (27.3 % vs. 54.5 %), alopecia (27.3 % vs. 50.0 %), epistaxis (22.7 % vs. 50.0 %), diarrhea (18.2 % vs. 50.0 %), and rash (13.6 % vs. 40.9 %) were lower among patients receiving T-DM1 + paclitaxel vs. T-DM1 + paclitaxel + pertuzumab.

**Table 2:** All-grade AEs (occurring in ≥20 %) or grade 3–4 AEs^a^ (occurring in >3 %) in phase 2a

Adverse event, n (%)	Grades 1 and 2		Grade 3		Grade 4		
	Group A (n = 22)	Group B (n = 22)	Group A (n = 22)	Group B (n = 22)	Group A (n = 22)	Group B (n = 22)	Total (N = 44)
Peripheral neuropathy	18 (81.8)	14 (63.6)	2 (9.1)	6 (27.3)	–	–	40 (90.9)
Fatigue	13 (59.1)	16 (72.7)	3 (13.6)	3 (13.6)	–	–	35 (79.5)
Nausea	9 (40.9)	9 (40.9)	1 (4.5)	1 (4.5)	–	–	20 (45.5)
Dry eye	6 (27.3)	11 (50.0)	–	1 (4.5)	–	–	18 (40.9)
Alopecia	6 (27.3)	11 (50.0)	–	–	–	–	17 (38.6)
Arthralgia	9 (40.9)	7 (31.8)	–	–	–	–	16 (36.4)
Epistaxis	5 (22.7)	11 (50.0)	–	–	–	–	16 (36.4)
Diarrhea	4 (18.2)	10 (45.5)	–	1 (4.5)	–	–	15 (34.1)
Thrombocytopenia	3 (13.6)	3 (13.6)	3 (14.6)	2 (9.1)	2 (9.1)	–	13 (29.5)
Decreased appetite	5 (22.7)	7 (31.8)	–	–	–	–	12 (27.3)
Neutropenia	1 (4.5)	–	5 (22.7)	3 (13.6)	2 (9.1)	1 (4.5)	12 (27.3)
Rash	2 (9.1)	9 (40.9)	1 (4.5)	–	–	–	12 (27.3)
Vision blurred	6 (27.3)	6 (27.3)	–	–	–	–	12 (27.3)
Myalgia	4 (18.2)	6 (27.3)	1 (4.5)	–	–	–	11 (25.0)
Dyspnea	3 (13.6)	7 (31.8)	–	–	–	–	10 (22.7)
Anemia	2 (9.1)	4 (18.2)	2 (9.1)	1 (4.5)	–	–	9 (20.5)
Constipation	4 (18.2)	4 (18.2)	1 (4.5)	–	–	–	9 (20.5)
Cough	4 (18.2)	5 (22.7)	–	–	–	–	9 (20.5)
Mucosal inflammation	1 (4.5)	4(18.2)	1 (4.5)	1 (4.5)	–	–	7 (15.9)
Muscular weakness	1 (4.5)	–	–	2 (9.1)	–	–	3 (6.8)
Decreased hemoglobin	–	–	–	1 (4.5)	1 (4.5)	–	2 (4.5)

*AE* adverse event

^a^No patient experienced a grade 5 AE

In phase 1b, 44 patients (73.3 %) discontinued paclitaxel due to AEs, but remained on other study medications. AEs leading to paclitaxel discontinuation in >1 patient were peripheral neuropathy (n = 25), neutropenia (n = 4), fatigue (n = 3), thrombocytopenia (n = 2), dry eye (n = 2), and hypertransaminasemia (n = 2). In phase 2a, 26 patients (59.1 %) discontinued paclitaxel due to AEs. Only peripheral neuropathy (n = 15; fatigue, n = 2) led to paclitaxel discontinuation in >1 patient. There were five deaths (phase 1b, n = 3; phase 2a, n = 2). Two deaths in phase 1b (sudden death and subdural hematoma) were considered treatment-related, while the third (pneumonia) was considered unrelated. Neither death in phase 2a (disease progression) was considered treatment-related.

### Feasibility

**Fig. 2:**
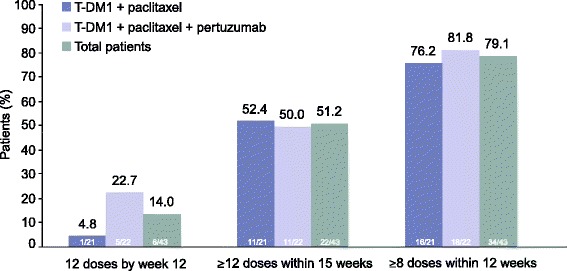
Feasibility of delivering weekly paclitaxel in combination with T-DM1 with or without pertuzumab. *T-DM1* trastuzumab emtansine

One phase 2a patient was nonevaluable for feasibility due to PD prior to receiving 12 doses of paclitaxel. Of the remaining 43 patients, 79.1 % (n = 34) received ≥8 paclitaxel doses within 12 weeks, 51.2 % (n = 22) received ≥12 doses within 15 weeks, and 14.0 % (n = 6) received 12 doses by week 12 (Fig. 2).

The median dose intensity of paclitaxel was 82.8 % (range, 52.1–101.3), and the median number of paclitaxel doses administered was 12 (1–48). The overall median treatment duration for paclitaxel in patients in Group A (no pertuzumab) and Group B (with pertuzumab) was 3.04 (range, 0.0–6.9) months and 2.55 (1.0 − 5.8) months, respectively. The median dose intensity of T-DM1 and pertuzumab was 95.9 % (52.2–104.5) and 100 % (67–100), respectively, and the median number of doses administered was 8.5 (1–14) and 8.5 (4–14), respectively.

### Efficacy

In phase 1b, 55 of 60 patients had measurable disease. The ORR was 54.5 % (95 % CI 40.6–68.0), and the CBR was 66.7 % (95 % CI 53.5–78.3) (Table 3). In phase 2a, 42 of 44 patients had measurable disease, and the overall ORR was 50.0 % (34.6–65.4) (Table 3). The ORR was 47.6 % (27.6–70.2) in those who did not receive pertuzumab (Group A) and was 52.4 % (29.8–72.4) in patients who did receive pertuzumab (Group B). The overall CBR for phase 2a was 56.8 % (41.6–71.0). The CBR was 54.5 % (32.7–74.0) in patients who did not receive pertuzumab (Group A) and was 59.1 % (38.3–79.3) in patients who did (Group B).

**Table 3:** Efficacy

	Phase 1b	Phase 2a		
	Total (N = 60)	Group A (n = 22)	Group B (n = 22)	All (N = 44)
Best overall response,^a^ n (%)				
Complete response	1 (2)	1 (4.8)^b^	1 (4.5)	2 (4.7)^c^
Partial response	34 (57)	13 (61.9)^b^	15 (68.2)	28 (65.1)^c^
Stable disease	22 (37)	6 (28.6)^b^	5 (22.7)	11 (25.6)^c^
Progressive disease	3 (5)	1 (4.8)^b^	1 (4.5)	2 (4.7)^c^
Clinical benefit rate^d^				
n (%)	40 (66.7)	12 (54.5)	13 (59.1)	25 (56.8)
95 % CI	53.5 − 78.3	32.7 − 74.0	38.3 − 79.3	41.6 − 71.0
No. of patients with measurable disease	n = 55	n = 21	n = 21	n = 42
Objective response rate^e^				
n (%)	30 (54.5)	10 (47.6)	11 (52.4)	21 (50.0)
95 % CI	40.6 − 68.0	27.6 − 70.2	29.8 − 72.4	34.6 − 65.4

*CI* confidence interval

^a^At any time point with responses ordered complete response > partial response > stable disease > progressive disease

^b^Of 21 patients with best response

^c^Of 43 patients with best response

^d^Includes patients with complete response, partial response, or stable disease of ≥6 months duration, as assessed by the investigator

^e^Includes only those patients with confirmed complete and partial responses, and is calculated based on patients with measurable disease

**Fig. 3:**
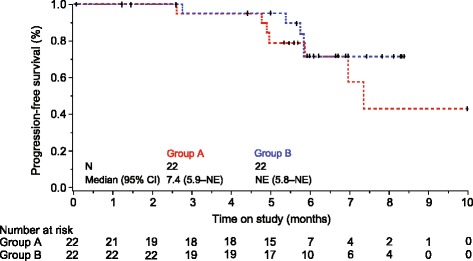
Investigator-assessed progression-free survival among phase 2a patients. *CI* confidence interval, *NE* not estimable. + censored due to withdrawal or enrollment in the extension study

The median duration of follow-up in phase 2a was 6.2 months. Median PFS for patients in Group A was 7.4 months (95 % CI 5.9–not estimable; Fig. 3). Median PFS was not reached in Group B.

### Pharmacokinetics

Pharmacokinetic analyses of T-DM1 conjugate, total trastuzumab, DM1, and paclitaxel suggested a low risk of drug − drug interactions between T-DM1 and paclitaxel (Additional file 4: Pharmacokinetics results, Tables S3–S5 and Figure S1).

### Biomarkers

Results from the exploratory biomarker analysis of HER2 mRNA are summarized in Additional file 5: Table S6. Due to small subgroup numbers, these data should be interpreted with caution.

## Discussion

This phase 1b/2a trial is the first to report on the use of T-DM1 combined with a taxane. Peripheral neuropathy was the most frequent all-grade AE in both study phases and was the main reason for paclitaxel discontinuation. Peripheral neuropathy is associated with taxane use [16, 17] and is less common with single-agent T-DM1 [18]. The rate of all-grade peripheral neuropathy in the present study (90.9 %) was similar to the 78–92 % incidence reported in studies of patients who were largely taxane-naive and administered trastuzumab + weekly paclitaxel 90 mg/m^2^ [19, 20]. This rate was also comparable to that observed in a phase 2 study of patients with MBC administered trastuzumab + pertuzumab + weekly paclitaxel 80 mg/m^2^ (82.1 %) [21]. In a phase 3 trial of patients with MBC administered trastuzumab + paclitaxel 175 mg/m^2^ q3w or 80 mg/m^2^ weekly, the incidence of grade 3 neuropathy was significantly greater in patients receiving weekly vs. q3w paclitaxel (24 % vs. 12 %, *P* = 0.0003) [17], further suggesting that this toxicity is largely paclitaxel-related. In the current study, the majority (81.8 %) of patients had received prior taxane treatment, which may have also contributed to the high rate of peripheral neuropathy.

In phase 2a, the incidence of all-grade fatigue in Group A (72.7 %) and Group B (86.4 %) was higher than that observed with single-agent T-DM1 in the phase 3 EMILIA (35.1 %) [2] and TH3RESA studies (25 %) [3], suggesting that this increase is due to the addition of paclitaxel and potentially pertuzumab. Rates of the most common grade ≥3 AEs in phase 2a, neutropenia (25.0 %) and peripheral neuropathy (18.2 %), were higher than reported for single-agent T-DM1 in the EMILIA (neutropenia, 2.0 %; peripheral neuropathy, <2 %) [2] and TH3RESA studies (neutropenia, 2.5 %; peripheral neuropathy, <2 %) [3]. However, the rate of grade ≥3 thrombocytopenia in phase 2a of our study (15.9 %) was similar to the incidence in EMILIA (12.9 %) [2], suggesting that thrombocytopenia was not potentiated by adding paclitaxel ± pertuzumab. In phase 2a of the present study, adding pertuzumab to T-DM1 + paclitaxel did not substantially alter toxicity, although rates of all-grade dry eye, alopecia, epistaxis, diarrhea, and rash were numerically higher in patients also receiving pertuzumab.

Overall, 51.2 % of phase 2a patients received ≥12 doses of weekly paclitaxel 80 mg/m^2^ within 15 weeks, and 14.0 % received 12 paclitaxel doses by week 12. The feasibility of combining T-DM1 and paclitaxel was lower than that reported in a phase 2 study of the first-line treatment of advanced breast cancer with trastuzumab + paclitaxel: 97 % (33/34) completed ≥12 weeks of treatment with weekly paclitaxel 90 mg/m^2^ + weekly trastuzumab [20]. This may have been due to the fact that most patients in our cohort had previously received taxane treatment; as mentioned, peripheral neuropathy, which is associated with taxane use [16, 17], was the most common AE leading to paclitaxel discontinuation.

The pharmacokinetics of T-DM1, total trastuzumab, and DM1 in this study were comparable with single-agent T-DM1. Moreover, the pharmacokinetics of paclitaxel were similar in the presence and absence of T-DM1, indicating that the potential for drug–drug interaction between T-DM1 and paclitaxel is low.

In phase 2a of our study, where patients had received a median of 5.0 systemic therapies in the metastatic setting, the ORR was 50.0 %, and the CBR was 56.8 %. Median PFS was not estimable due to limited follow-up of all patients.

The rationale for this study was based on pre-clinical data suggesting that taxanes could enhance the activity of T-DM1. We had assumed that combining T-DM1 with a conventional, non-targeted, cytotoxic agent such as paclitaxel would add to the toxicity of single-agent T-DM1, therefore negating the favorable tolerability profile conferred by the targeted mechanism of action of T-DM1, but the extent of this potentially increased toxicity was unclear. Neurotoxicity is a known treatment-limiting toxicity for weekly paclitaxel [19]. In our study, all-grade neurotoxicity occurred in 90.9 % of patients and grade 3–4 neurotoxicity occurred in 18.2 %; toxicity (predominately peripheral neuropathy) led to 48.8 % of patients being unable to receive ≥12 paclitaxel doses within 15 weeks and 86.0 % being unable to receive 12 paclitaxel doses by week 12. As the majority of patients on this study (phase 1b, 90 %; phase 2a, 82 %) had previously received taxane treatment, the high rate of peripheral neuropathy is not unexpected: taxane retreatment can lead to cumulative toxicities or exacerbation of chronic toxicities such as neuropathy [22]. Thus, it is possible that this regimen may be better tolerated in those who are taxane-naive. In light of the clinical activity of this regimen and given that the primary reason for treatment discontinuation was neuropathy, evaluation of this regimen in patients without prior taxane exposure may be warranted. Alternatively, an intermittent vs. weekly paclitaxel schedule may increase the feasibility of this regimen in those who are taxane-experienced.

## Conclusions

With its clinical activity, as demonstrated by an ORR of 50.0 % and a CBR of 56.8 %, these data suggest that there is potential for T-DM1 to be combined with paclitaxel and pertuzumab. However, in this pre-treated population, rates of peripheral neuropathy were high and resulted in frequent and early discontinuation of paclitaxel. It is unclear whether adding pertuzumab or paclitaxel, or the combination of both agents, adds to the substantial clinical activity of single-agent T-DM1 in patients with previously treated advanced breast cancer.

## Additional files

Additional file 1: Table S1. Dose-limiting toxicity criteria. (DOC 136 kb) Additional file 2: Supplemental methods. Patient exclusion criteria, pharmacokinetic analysis methods, and biomarker analysis methods. (DOC 138 kb) Additional file 3: Table S2. All-grade AEs (occurring in ≥20 %) or grade ≥3 AEs (occurring in >3 %) of the total patient population in phase 1b. (PDF 72 kb) Additional file 4: Pharmacokinetics results. Supplemental results for pharmacokinetics analysis. **Table S3:** Summary of pharmacokinetic exposure of paclitaxel in cycle 1 (absence of T-DM1) and cycle 2 (presence of T-DM1). **Table S4:** Summary of pharmacokinetic exposure of T-DM1-related analytes (serum T-DM1, serum total trastuzumab and plasma DM1) in cycle 1 following q3w dosing. **Table S5:** Summary of pharmacokinetic exposure of T-DM1-related analytes (serum T-DM1, serum total trastuzumab and plasma DM1) in cycle 1 following qw dosing. **Figure S1:** Mean paclitaxel concentration time profiles at (A) a dose of 65 mg/m^2^ and (B) a dose of 80 mg/m^2^. (DOC 732 kb) Additional file 5: Table S6. Summary of ORR by HER2 mRNA expression level as measured by quantitative reverse transcription polymerase chain reaction. (DOC 138 kb)

## Abbreviations

AE: Adverse events
ALP: Alkaline phosphatase
ALT: Alanine transaminase
AST: Aspartate aminotransferase
AUC: Area under plasma concentration-time curve
CBR: Clinical benefit rate
CI: Confidence interval
CL: Clearance
C_max_: Peak plasma concentration
CR: Complete response
DLT: Dose-limiting toxicity
ECOG: Eastern Cooperative Oncology Group
ER: Estrogen receptor
HER2: Human epidermal growth factor receptor 2
HR: Hazard ratio
LABC: Locally advanced breast cancer
LC-MS/MS: Liquid chromatography-tandem mass spectrometry
LD: Loading dose
LLOQ: Lower limit of quantitation
MBC: Metastatic breast cancer
MQC: Minimum quantifiable concentration
MTD: Maximum tolerated dose
MUGA: Multigated acquisition
NCA: Noncompartmental analysis
NE: Not estimable
ORR: Objective response rate
OS: Overall survival
PD: Progressive disease
PFS: Progression-free survival
PR: Partial response
PR: Progesterone receptor
q3w: Every three weeks
RECIST: Response Evaluation Criteria in Solid Tumors
SD: Stable disease
t_1/2_: Elimination half-life
T-DM1: Trastuzumab emtansine
ULN: Upper limit of normal
V_ss_: Volume of distribution at steady state
