# Supplementary material for: Phase 1b/2a study of trastuzumab emtansine (T-DM1), paclitaxel, and pertuzumab in HER2-positive metastatic breast cancer
Source: Breast Cancer Res. 2016 Mar 15;18:34. doi: 10.1186/s13058-016-0691-7 (PMC4791863; doi:10.1186/s13058-016-0691-7)
Supplement: Additional file 1: Table S1. — Dose-limiting toxicity criteria. (DOC 136 kb) [file 13058_2016_691_MOESM1_ESM.doc]

**Additional file 1**

**Table S1 Dose-limiting toxicity criteria**

| Initial DLT criteria | Revised DLT criteria |
| --- | --- |
| Any grade ≥3 nonhematologic AE not due to disease progression^a^ | Any grade ≥4 nonhematologic AE or any grade 3 nonhematologic AE not improving to baseline value or grade ≤1 by day 21 from treatment |
| Any grade ≥3 elevation of serum bilirubin, ALT, AST, or ALP lasting >72 hours^b^ | Any grade ≥3 elevation of serum bilirubin, >10 × ULN elevation of ALT, AST, or ALP or any >5–10 × ULN elevation not improving to ≤5 × ULN (grade ≤2) by day 7 |
| Grade ≥4 thrombocytopenia | Grade 4 thrombocytopenia or grade 3 thrombocytopenia not recovered to grade ≤2 by day 7 |
| Grade ≥4 neutropenia lasting >72 hours or accompanied by a fever | Grade 4 neutropenia lasting ≥7 days or febrile neutropenia |
| Any subjectively intolerable toxicity felt by the investigator to be related to T-DM1, paclitaxel, or pertuzumab |  |
| Any treatment-related toxicity prompting a dose delay or modification of T-DM1 during the DLT observation period | Any treatment-related toxicity prompting a dose reduction of T-DM1 during the DLT observation period^c^ |
| Any treatment-related toxicity prompting more than 1 weekly paclitaxel dose delay or any weekly paclitaxel dose modification during the DLT observation period^d^ |  |

*AE* adverse event, *ALP* alkaline phosphatase, *ALT* alanine transaminase, *AST* aspartate aminotransferase, *DLT* dose-limiting toxicity, *T-DM1* trastuzumab emtansine, *ULN* upper limit of normal

^a^Except alopecia (any grade), grade 3 diarrhea that responds to therapy, grade 3 nausea or vomiting in absence of premedication that responds to therapy.

^b^For patients with grade 2 hepatic transaminase levels at baseline (≤5 × ULN) as a result of liver or bone metastases; ALT, AST, or ALP levels ≥10 × ULN was considered a DLT.

^c^A missed dose of T-DM1 during the observation period was not considered a DLT.

^d^One weekly paclitaxel dose delay during the DLT observation period was not considered a DLT.
