# Supplementary material for: Phase 1b/2a study of trastuzumab emtansine (T-DM1), paclitaxel, and pertuzumab in HER2-positive metastatic breast cancer
Source: Breast Cancer Res. 2016 Mar 15;18:34. doi: 10.1186/s13058-016-0691-7 (PMC4791863; doi:10.1186/s13058-016-0691-7)
Supplement: Additional file 2: — Supplemental methods. Patient exclusion criteria, pharmacokinetic analysis methods, and biomarker analysis methods. (DOC 138 kb) [file 13058_2016_691_MOESM2_ESM.doc]

**Additional file 2**

**SUPPLEMENTAL METHODS**

**Patients**

Exclusion criteria included prior treatment with T-DM1 or pertuzumab; exposure to cumulative anthracycline doses of doxorubicin >500 mg/m^2^ (or the equivalent); grade ≥2 peripheral neuropathy (phase 1b only) or any-grade peripheral neuropathy (phase 2a only); history of clinically significant cardiac dysfunction; brain metastases that were untreated, progressive, or required therapy to control symptoms within 60 days of study treatment; <21 days since last anti-tumor therapy; and a history of intolerance or hypersensitivity to trastuzumab or paclitaxel and/or adverse events (AEs) related to trastuzumab or paclitaxel that resulted in treatment discontinuation.

**Pharmacokinetic analysis**

In phase 1b, plasma samples were drawn on day –1 of cycle 1 (preinfusion, within 0.25 hours postinfusion, and at 1, 2, 4, 6, and 24 hours postinfusion) following a single dose of paclitaxel to determine the pharmacokinetics of paclitaxel in the absence of T-DM1. Additional blood samples were taken on day 1 of cycle 2, using the same sampling schedule. Serum T-DM1 and total trastuzumab levels were analyzed by validated ELISA methods at Genentech, Inc, with a minimum quantifiable concentration (MQC) of 40 ng/mL for both analytes. Free DM1 concentrations in anticoagulant (lithium heparin)-exposed plasma were determined by Xendo Drug Development B.V. (Groningen, The Netherlands) using a validated electrospray liquid chromatography-tandem mass spectrometry (LC-MS/MS) method, with a lower limit of quantitation (LLOQ) following tris(2-carboxyethyl)phosphine reduction of 1.00 nM (0.737 ng/mL). Plasma paclitaxel concentrations were analysed by a validated LC-MS/MS method, with a LLOQ of 2 ng/mL.

To assess the effects of T-DM1 on paclitaxel pharmacokinetics, paclitaxel pharmacokinetic parameters (peak plasma concentration [C_max_], time to reach C_max_ [T_max_], elimination half-life [t_1/2_], area under plasma concentration-time curve from time 0 to infinity [AUC_inf_], clearance [CL], and apparent volume of distribution at steady state [V_ss_]) were compared between cycle 1 (absence of T-DM1) and cycle 2 (presence of T-DM1). To assess the potential impact of paclitaxel on the pharmacokinetics of T-DM1, serum samples were taken following administration of T-DM1 (preinfusion and at 0.25 hours, 4 hours, and 7 days postinfusion) on day 1 of cycle 1 and following administration of paclitaxel and T-DM1 on day 1 of cycle 2. Pharmacokinetic parameters for T-DM1 and DM1 after the first dose were estimated based on non-compartmental analysis (NCA) using WinNonlin® 5.1 hosted by the Pharsight® Knowledgebase Server™ and compared against historical estimates from a single-arm study that also explored T-DM1 (0.3 mg/kg to 4.8 mg/kg) [1].

**Biomarker analysis**

The biomarker analysis was performed at Targos Molecular Pathology GmBH (Kassel, Germany). *HER2* mRNA levels were determined via quantitative real time-polymerase chain reaction (cobas®, Roche Molecular Diagnostics) in all patients who provided informed consent and had available samples. *G6PDH* served as a reference. Subgroups were stratified about the distributed median *HER2* mRNA values for all phase 1b and phase 2a participants with available data (ie, above the median value [>median] or at or below the median value [≤median]). Data from phase 1b and phase 2a were examined separately and in combination. Data from phase 2a participants were also stratified by treatment arm (ie, Group A and Group B).

**Reference**

1. Krop IE, Beeram M, Modi S, Jones SF, Holden SN, Yu W, et al. Phase I study of trastuzumab-DM1, an HER2 antibody-drug conjugate, given every 3 weeks to patients with HER2-positive metastatic breast cancer. J Clin Oncol. 2010;28:2689–704.
