# Supplementary material for: Phase 1b/2a study of trastuzumab emtansine (T-DM1), paclitaxel, and pertuzumab in HER2-positive metastatic breast cancer
Source: Breast Cancer Res. 2016 Mar 15;18:34. doi: 10.1186/s13058-016-0691-7 (PMC4791863; doi:10.1186/s13058-016-0691-7)
Supplement: Additional file 3: Table S2. — All-grade AEs (occurring in ≥20 %) or grade ≥3 AEs (occurring in >3 %) of the total patient population in phase 1b. (PDF 72 kb) [file 13058_2016_691_MOESM3_ESM.pdf]

Additional file 3

Table S2 All-grade AEs (occurring in ≥20%) or grade ≥3 AEs[a] (occurring in >3%) of the total patient population in phase 1b

	Regimen 1 T-DM1 q3w + paclitaxel qw (n=26), n (%)		Regimen 2 T-DM1 q3w + paclitaxel qw + pertuzumab q3w (n=10), n (%)		Regimen 3 T-DM1 qw + paclitaxel qw (n=21), n (%)		Regimen 4 T-DM1 qw + paclitaxel qw + pertuzumab q3w (n=3), n (%)		All patients (N=60)
	Grade 1/2	Grade ≥3	Grade 1/2	Grade ≥3	Grade 1/2	Grade ≥3	Grade 1/2	Grade ≥3	All grades
Peripheral neuropathy	16 (61.5)	7 (26.9)	6 (60.0)	2 (20.0)	16 (76.2)	4 (20.0)	2 (66.7)	1 (33.3)	54 (90.0)
Fatigue	19 (73.1)	4 (15.4)	8 (80.0)	–	12 (57.1)	6 (28.6)	1 (33.3)	1 (33.3)	51 (85.0)
Epistaxis	9 (34.6)	–	6 (60.0)	–	9 (42.9)	–	3 (100)	–	27 (45.0)

Alopecia	10 (38.5)	–	6 (60.0)	–	8 (38.1)	–	1 (33.3)	–	25 (41.7)
Diarrhoea	7 (26.9)	1 (3.8)	4 (40.0)	1 (10.0)	7 (33.3)	1 (4.8)	2 (66.7)	–	23 (38.3)
Dry eye	6 (23.1)	2 (7.7)	3 (30.0)	–	9 (42.9)	–	2 (66.7)	–	22 (36.7)
Nausea	8 (30.8)	–	3 (30.0)	–	10 (47.6)	–	–	–	21 (35.0)
Arthralgia	8 (30.8)	–	3 (30.0)	–	6 (28.6)	1 (4.8)	2 (66.7)	–	20 (33.3)
Rash	5 (19.2)	–	7 (70.0)	–	6 (28.6)	–	2 (66.7)	–	20 (33.3)
Hypokalemia	3 (11.5)	2 (7.7)	2 (20.0)	1 (10.0)	3 (14.3)	4 (19.0)	1 (33.3)	–	16 (26.7)
Decreased appetite	7 (26.9)	–	1 (10.0)	–	8 (38.1)	–	–	–	16 (26.7)
Constipation	8 (30.8)	–	3 (30.0)	–	3 (14.3)	–	1 (33)	–	15 (25.0)
Cough	8 (30.8)	–	2 (20.0)	–	4 (19.0)	–	1 (33.3)	–	15 (25.0)
Upper respiratory tract infection	8 (30.8)	–	2 (20)	–	4 (19.0)	–	1 (33.3)	–	15 (25.0)

Vision blurred	4 (15.4)	1 (3.8)	3 (30.0)	–	5 (23.8)	1 (4.8)	1 (33.3)	–	15 (25.0)
Anaemia	2 (7.7)	4 (15.4)	2 (20.0)	–	2 (9.5)	3 (14.3)	–	1 (33.3)	14 (23.3)
Dyspnoea	6 (23.1)	–	1 (10.0)	–	6 (28.6)	–	1 (33.3)	–	14 (23.3)
Neutropenia	–	3 (11.5)	–	3 (30.0)	2 (9.5)	5 (23.8)	–	1 (33.3)	14 (23.3)
Dry Mouth	5 (19.2)	–	1 (10.0)	–	6 (28.6)	–	1 (33.3)	–	13 (21.7)
Thrombocytopenia	2 (7.7)	2 (7.7)	1 (10.0)	1 (10.0)	2 (9.5)	2 (9.5)	–	1 (33.3)	11 (18.3)
Cellulitis	1 (3.8)	2 (7.7)	2 (20.0)	–	1 (4.8)	2 (9.5)	–	–	9 (15.0)
Abdominal pain	2 (7.7)	2 (7.7)	–	–	1 (4.8)	1 (4.8)	–	–	6 (10.0)
Alanine aminotransferase increased	1 (3.8)	1 (3.8)	–	1 (10.0)	2 (9.5)	–	–	–	5 (8.3)
Leukopenia	2 (7.7)	1 (3.8)	–	1 (10.0)	1 (4.8)	–	–	–	5 (8.3)

Pneumonia	–	–	–	2 (20.0)	–	2[b] (9.5)	–	–	4 (6.7)
Hyponatremia	–	2 (7.7)	–	–	2 (9.5)	–	–	–	4 (6.7)
Hypertransaminasemia	–	–	–	1 (10.0)	1 (4.8)	–	–	1 (33.3)	3 (5.0)
Gastroenteritis	–	1 (3.8)	–	1 (10.0)	–	–	–	–	2 (3.3)
Pneumonitis	–	1 (3.8)	–	–	–	1 (4.8)	–	–	2 (3.3)
Sepsis	–	–	–	–	–	2 (9.5)	–	–	2 (3.3)

AE adverse event, *qw* weekly, *q3w* every 3 weeks, *T-DM1* trastuzumab emtansine

[a]There were 3 grade 5 events: sudden death (regimen 1), pneumonia (regimen 3), and acute subdural hematoma (regimen 3).

[b]One event was grade 5.
