# Supplementary material for: Phase 1b/2a study of trastuzumab emtansine (T-DM1), paclitaxel, and pertuzumab in HER2-positive metastatic breast cancer
Source: Breast Cancer Res. 2016 Mar 15;18:34. doi: 10.1186/s13058-016-0691-7 (PMC4791863; doi:10.1186/s13058-016-0691-7)
Supplement: Additional file 4: — Pharmacokinetics results. Supplemental results for pharmacokinetics analysis. Table S3: Summary of pharmacokinetic exposure of paclitaxel in cycle 1 (absence of T-DM1) and cycle 2 (presence of T-DM1). Table S4: Summary of pharmacokinetic exposure of T-DM1-related analytes (serum T-DM1, serum total trastuzumab and plasma DM1) in cycle 1 following q3w dosing. Table S5: Summary of pharmacokinetic exposure of T-DM1-related analytes (serum T-DM1, serum total trastuzumab and plasma DM1) in cycle 1 following qw dosing. Figure S1: Mean paclitaxel concentration time profiles at (A) a dose of 65 mg/m2 and (B) a dose of 80 mg/m2. (DOC 732 kb) [file 13058_2016_691_MOESM4_ESM.doc]

**Additional file 4**

**Pharmacokinetics results**

Mean paclitaxel pharmacokinetic profiles by nominal time were similar in the absence (cycle 1) and presence (cycle 2) of T-DM1 for each paclitaxel dose (Figure S1 and Table S3). The paclitaxel CL and V_ss_ values identified in the current study are similar to those reported in the literature (19.0 ± 3.8 L/h/m^2^ and 158 ± 47.4 L/m^2^, respectively) [1]. Moreover, paired t-tests to compare paclitaxel AUC_inf_ in the absence (cycle 1) and presence of T-DM1 (cycle 2) within individual patients yielded *P*-values >0.05 for paclitaxel 65 mg/m^2^ (n=24, *P*=0.926) and paclitaxel 80 mg/m^2^ (n=17, *P*=0.996). These results suggest no impact of T-DM1 on the pharmacokinetics of paclitaxel.

Mean T-DM1 exposure parameters by NCA are listed in Table S4 and Table S5 for the every 3 weeks (q3w) and weekly (qw) regimens in the presence of paclitaxel.

Mean T-DM1 conjugate C_max_ values, which were attained for most patients within 0.25 hours postinfusion, increased with dose and ranged from 24.3–87.8 μg/mL across the dose levels tested. Overall, the observed serum mean C_max_ values of T-DM1 conjugate across all tested dose levels for the q3w and qw regimens were largely comparable with those reported in a historical single-agent study of T-DM1 [2]. As differences in pharmacokinetic sampling points between the current study and the aforementioned historical single-agent study may have confounded the comparison of T-DM1 conjugate AUC_0-7_ and AUC_0-21_, a population pharmacokinetic analysis was instead used for cross-study comparison. T-DM1 conjugate post-hoc pharmacokinetic parameters (CL and central volume of distribution) were within the range of values previously reported for single-agent T-DM1 [3-5], suggesting no effect of concomitant paclitaxel on T-DM1 conjugate pharmacokinetics.

Plasma DM1 C_max_ values after the first dose of T-DM1 were <7 ng/mL for T-DM1 + paclitaxel. At most timepoints, DM1 concentrations were close to or below the LLOQ value of 0.737 ng/mL. These results are similar to single-agent historical data [2] and suggest that paclitaxel has no impact on DM1 exposure. Collectively, these data indicate that there is a low risk of pharmacokinetic drug interactions when T-DM1 and paclitaxel are administered concomitantly in patients with HER2-positive MBC.

Because study patients had received prior trastuzumab treatment, 15 of 26 patients in the T-DM1 q3w cohorts and 14 of 21 patients in the T-DM1 qw cohorts had measurable trastuzumab concentrations before first T-DM1 infusion. The presence of serum trastuzumab prior to T-DM1 dosing in a subset of patients contributed to the high variability observed in pharmacokinetic parameters for total trastuzumab, which was the sum of T-DM1 and unconjugated trastuzumab. Mean C_max_ values for total trastuzumab ranged from 39.9**–**97.6 μg/mL across the dose levels tested. The mean C_max_ and AUC values for total trastuzumab were higher than those of T-DM1. A cross-study comparison of total trastuzumab concentrations was not performed, as it would have likely been confounded by the variability in baseline trastuzumab levels.

**References**

1. Paclitaxel package insert, Bristol-Myers Squibb, Princeton, NJ, 2007.

2. Krop IE, Beeram M, Modi S, Jones SF, Holden SN, Yu W, et al. Phase I study of trastuzumab-DM1, an HER2 antibody-drug conjugate, given every 3 weeks to patients with HER2-positive metastatic breast cancer. J Clin Oncol. 2010;28:2689–704.

3. Lu D, Modi S, Elias A Agarwal P, Yi J-H, Guardino AE, Althaus B, Girish S. Pharmacokinetics (PK) of trastuzumab emtansine and paclitaxel or docetaxel in patients with HER2-positive metastatic breast cancer (MBC) previously treated with a trastuzumab-containing regimen. Presented at: 34th Annual San Antonio Breast Cancer Symposium. San Antonio, TX, USA, 6–10 December 2011.

4. Lu D, Joshi A, Agarwal P, Wang B, LoRusso P, Martín M, et al. Assessment of drug interaction potential of an antibody drug conjugate with other therapeutic agents: case studies of trastuzumab emtansine (T-DM1) in combination with pertuzumab or taxane. Presented at: American Society of Clinical Pharmacology and Therapeutics Annual Meeting. National Harbor, MD, USA, 12–17 March 2012.

5. Lu D, Sahasranaman S, Zhang Y, Girish S. Strategies to address drug interaction potential for antibody–drug conjugates in clinical development Bioanalysis. 2013;5: 1115–30.

**Table S3 Summary of pharmacokinetic exposure of paclitaxel in cycle 1 (absence of T-DM1) and cycle 2 (presence of T-DM1). Data are presented as mean ± standard deviation**

| Dose  (mg/m^2^) | Cycle | N | C_max_ (ng/mL) | AUC_inf_ (hr·ng/mL) | t_1/2_ (hr) | CL (L/hr/m^2^) | V_ss_ (L/m^2^) |
| --- | --- | --- | --- | --- | --- | --- | --- |
| 65 | 1 | 29 | 1430±53.5 | 3440±32.3 | 9.89±17.1 | 20.7±31.1 | 167±56.3 |
|  | 2 | 24 | 1280±59.8 | 3520±38 | 11.7 ±25.1 | 21±36.4 | 220±57.3 |
| 80 | 1 | 18 | 1540±64.4 | 3890±48 | 8.94±20 | 22.8±51.1 | 166±78.9 |
|  | 2 | 17 | 1590±56.7 | 4220±49.8 | 10.8±30.8 | 23±44.1 | 196±56.5 |

*AUC_inf_* area under plasma concentration-time curve from time 0 to infinity, *CL* clearance, *C_max_* peak plasma concentration, *t_1/2_* elimination half-life, *V_ss_* volume of distribution at steady state

**Table S4** Summary of pharmacokinetic exposure of T-DM1-related analytes (serum T-DM1, serum total trastuzumab and plasma DM1) in cycle 1 following q3w dosing (mean ± standard deviation)

|  | Cohort | A | B | 1 | J | D | 3B |
| --- | --- | --- | --- | --- | --- | --- | --- |
|  | Dose (mg/kg) | 2 | 2 | 2.4 | 2.4 | 3 | 3.6 |
| T-DM1 | C_max_ (µg/mL) | 53.5 ±3.11 (n=2) | 44.9 ±4.8 (n=6) | 43.6 ±16.3 (n=8) | 54.6 ±13.6 (n=3) | 62.2 ±18.9 (n=3) | 76.1 ±31.5 (n=3) |
|  | AUC_0-21_ (day·µg/mL) | 241 ±57.2 (n=2) | 216 ±118 (n=6) | 264 ±77.4 (n=6) | 271 ±51.2 (n=3) | 382 ±140 (n=3) | 523 ±288 (n=3) |
| Total Trastuzumab | C_max_ (µg/mL) | 73.9 ±33.4 (n=2) | 61 ±23.2 (n=6) | 62 ±15.2 (n=8) | 55.2 ±12.3 (n=3) | 91 ±45.4 (n=3) | 87.4 ±39.5 (n=3) |
|  | AUC_0-21_ (day·µg/mL) | 633 ±496 (n=2) | 518 ±359 (n=6) | 460 ±308 (n=6) | 401 ±249 (n=3) | 959 ±609 (n=3) | 764 ±372 (n=3) |
| DM1 | C_max_^a^ (ng/mL) | NA | 2.02-2.69 (n=4) | 2.00-4.89 (n=8) | 2.62-2.76 (n=3) | 2.60-4.09 (n=3) | 3.16-6.31 (n=3) |

*AUC* area under plasma concentration-time curve, *C_max_* peak plasma concentration, *q3w* every 3 weeks, *T-DM1* trastuzumab emtansine

^a^Due to the low levels of DM1, the range of C_max_ in cycle 1 is reported.

**Table S5** Summary of pharmacokinetic exposure of T-DM1-related analytes (serum T-DM1, serum total trastuzumab and plasma DM1) in cycle 1 following qw dosing (mean ± standard deviation)

|  | Cohort | 6 | 7 | 8 | F | 4 |
| --- | --- | --- | --- | --- | --- | --- |
|  | Dose (mg/kg) | 1.2 | 1.6 | 2 | 2.4 | 2.4 |
| T-DM1 | C_max_ (µg/mL) | 24.3 ±2.61 (n=3) | 26 ±11.2 (n=9) | 39.4 ±7.09 (n=3) | 63.7 ±8.72 (n=3) | 87.8 ±75.3 (n=3) |
|  | AUC_0-7_ (day·µg/mL) | 84.6 ±15.2 (n=3) | 91.9 ±41.8 (n=8) | 150 ±28.3 (n=3) | 242 ±18 (n=3) | 165 ±42.4 (n=3) |
| Total Trastuzumab | C_max_ (µg/mL) | 57.8 ±31.7 (n=3) | 39.9 ±24.1 (n=9) | 66.1 ±25.2 (n=3) | 97.6 ±45.7 (n=3) | 92.1 ±69.4 (n=3) |
|  | AUC_0-7_ (day·µg/mL) | 275 ±170 (n=3) | 149 ±94.1 (n=8) | 295 ±121 (n=3) | 460^a^ ±250 (n=3) | 270 ±62.2 (n=3) |
| DM1 | C_max_^b^ (ng/mL) | 0.78– 1.16 (n=3) | 0.75 – 2.67 (n=7) | 2.56 – 3.77 (n=3) | 2.23 – 3.17 (n=3) | 2.41 – 6.90 (n=3) |

*AUC* area under plasma concentration-time curve, *C_max_* peak plasma concentration, *qw* weekly, *T-DM1* trastuzumab emtansine

^a^One patient had a much higher predose total trastuzumab level in cycle 1 (~67.2 ug/mL).

^b^Due to the low levels of DM1, the range of C_max_ in cycle 1 is reported.

**Fig. S1** Mean paclitaxel concentration time profiles at (A) a dose of 65 mg/m^2^ and (B) a dose of 80 mg/m^2^. Error bars represent standard deviation.

A.

B.
