# Supplementary material for: Phase 1b/2a study of trastuzumab emtansine (T-DM1), paclitaxel, and pertuzumab in HER2-positive metastatic breast cancer
Source: Breast Cancer Res. 2016 Mar 15;18:34. doi: 10.1186/s13058-016-0691-7 (PMC4791863; doi:10.1186/s13058-016-0691-7)
Supplement: Additional file 5: Table S6. — Summary of ORR by HER2 mRNA expression level as measured by quantitative reverse transcription polymerase chain reaction. (DOC 138 kb) [file 13058_2016_691_MOESM5_ESM.doc]

**Additional file 5**

**Table S6** Summary of ORR by HER2 mRNA expression level as measured by quantitative reverse transcription polymerase chain reaction

|  | Phase 1b  total  (N=60) | Phase 2a | | | Phase 1b/2a  total  (N=104) |
| --- | --- | --- | --- | --- | --- |
|  |  | Group A (n=22) | Group B  (n=22) | All  (N=44) |  |
| TDM4652g Study | | | | | |
| Patients with measurable disease, n | 55 | 21 | 21 | 42 | 97 |
| ORR^a^, n (%)  95% CI | 30 (54.5)  40.6−68.0 | 10 (47.6)  27.6−70.2 | 11 (52.4) 29.8−72.4 | 21 (50.0) 34.6−65.4 | 51 (52.6)  42.2−62.8 |
| Subset of patients with HER2 mRNA data | | | | |  |
| HER2 mRNA ≤ median^b^, n^c^  ORR^a^, n (%)  95% CI | 17  7 (41.2)  18.4–66.3 | 8  4 (50.0)  19.3–80.7 | 8  3 (37.5)  11.1–71.1 | 16  7 (43.8)  19.8–70.1 | 33  14 (42.4)  26.8–59.9 |
| HER2 mRNA >median^b^, n^c^  ORR^a^, n (%)  95% CI | 21  13 (61.9)  39.8–80.3 | 6  2 (33.3)  (6.3–72.9) | 5  4 (80.0)  (34.3–99.0) | 11  6 (54.5)  (25.0–80.0) | 32  19 (59.4)  (42.0–76.9) |

*CI* confidence interval, *HER2* human epidermal growth factor receptor 2, *mRNA* messenger RNA, *ORR* objective response rate

^a^Includes only those patients with confirmed complete and partial responses and is calculated based on the number of patients with measurable disease.

^b^The median value is 12.25, which was calculated based on all patients who had specimen analyzed and reported for HER2 mRNA across all treatments in phase 1b/2a.

^c^Number of patients with measurable disease and with HER2 mRNA values.
